# The World Heart Observatory: Harnessing the Power of Data for Cardiovascular Health

**DOI:** 10.5334/gh.1126

**Published:** 2022-06-06

**Authors:** Pablo Perel, Oana Scarlatescu, Alvaro Avezum, Richard A. Chazal, Mark A. Creager, Jagat Narula, Daniel Pineiro, Dorairaj Prabhakaran, Fernando Wyss, Huo Yong, Liesl Zühlke, Jean-Luc Eisele, Fausto Pinto

**Affiliations:** 1 World Heart Federation, Geneva, CH; 2 London School of Hygiene & Tropical Medicine, UK; 3 International Research Center, Hospital Alemão Oswaldo Cruz & Brazilian Cardiology Society, São Paulo, Brazil; 4 Heart and Vascular Institute, Lee Health, Fort Myers, Florida, USA; 5 Dartmouth-Hitchcock Medical Center, Geisel School of Medicine at Dartmouth, Lebanon, NH, USA; 6 Mount Sinai Hospitals, New York City, New York, USA; 7 Public Health Foundation of India, Haryana, IN; 8 Interamerican Society of Cardiology – SIAC, Guatemala City, GT; 9 Peking University First Hospital, Beijing, CN; 10 South African Medical Research Council and University of Cape Town, Cape Town, SA

**Keywords:** World Heart Observatory, Data for cardiovascular health, Data Storytelling, Data Advocacy

Cardiovascular disease (CVD) is the world’s number one cause of death, claiming an estimated 18.6 million lives in 2019 [1]. That statistic is staggering. Most importantly, an estimated 80 percent of deaths from heart disease and stroke could be avoided with early interventions that span the continuum from education and prevention to diagnosis and treatment. As the COVID-19 pandemic has proven, health data is a powerful tool for use across the continuum of care. Some initiatives, such as Our World in Data [2], demonstrated the potential for the use of data to understand the spread of disease, spot emerging trends, identify the most vulnerable populations, and evaluate the impact of various clinical and public health interventions.

Data and information are key to understanding needs, trends, causes, responses, and prediction in global cardiovascular health. Accurate information helps policymakers, researchers and programme implementers grasp the true burden of cardiovascular disease and its risk factors, understand why certain populations are affected differently by the same disease, identify missed opportunities for the detection and management of cardiovascular risk at population and health system levels, obtain insight into best interventions, plan resources, and monitor progress.

Valuable data initiatives for CVD and other non-communicable diseases exist at global, national, and regional levels, including the NCD Risk Factor Collaboration [3], the Global Burden of Disease [4], the PURE Study [5], the Prospective Studies Collaboration [6], and many others are conducted by World Heart Federation Members [7,8,9,10,11,12]. Indeed, the world has never had more data initiatives. There is, however, insufficient coordination and alignment among them, as data projects span different domains, platforms, organizations, and methodological approaches. There is thus a need for these data initiatives to be mapped, collated, and curated to transform health data into actionable knowledge.

The World Heart Federation has a unique opportunity to mobilize the cardiovascular health community around the different data initiatives and leverage the current digital revolution. Building on the strength of its +200 Members around the world, on its official relations with the World Health Organization, and on its mission to translate science into policy, WHF launched the World Heart Observatory on 1 February 2022 as a collaborative platform that collates high-quality data from different sources to provide the most reliable information related to cardiovascular conditions, risk factors, and interventions.

The World Heart Observatory reveals how the burden of cardiovascular disease has changed over the years, the global and regional impact, causes for expansion of disease, and insight into progress within and between countries in prevention and control. The World Heart Observatory relies on strong data science, strong communication, *and calls for strong advocacy*. In practice, this translates to accurate data from multiple sources, conveyed through compelling storytelling and visualisations to contextualize information that is relevant for policymakers, researchers, clinicians, health advocates and people living with cardiovascular disease.

The World Heart Observatory works in collaboration with a multitude of partners in academia (The Institute for Health Metrics and Evaluation, University of Essex), WHF Member organizations, international organizations (e.g., World Health Organization), civil society and the private sector (e.g. foundations) as it grows the platform that will provide several functions including:

1. An online hub for existing but disconnected data initiatives in the cardiovascular health space, including WHF’s data projects such as the CVD Scorecards, Roadmap Surveys, and the Global Study on COVID-19 and CVD.
2. An open, searchable dashboard of WHF Members’ data projects.
3. A driver of capacity building around data in cardiovascular health.
4. A platform to map and visualise gaps in data and identify research needs.
5. A builder of data capacity among national and regional health organizations in the cardiovascular arena.
6. An easy-to-use resource for educators, researchers, patients, and policy makers.

As an ambitious and constantly evolving initiative, which aims to be a data broker and data steward, the World Heart Observatory will encounter challenges. There will be a need to assess and make decisions about data accuracy and of the underpinning methodologies, including complex modelling. It will be crucial to understand how to deal with incomplete data on morbidity and mortality in many countries and find ways to obtain reliable data everywhere including low-income settings. The Observatory must help make sense of large data sets and put data of variable quality into context for decision makers and in calls to action for better cardiovascular health. Also, it will provide particular attention to areas where data is scarce or of limited quality and identify strategies to improve this. Finally, it will promote new collaborations and data initiatives with collaborating organisations. In doing so, the Observatory strives to help fulfil the Mission of the World Heart Federation: To stimulate and promote the exchange of information, ideas, practices across all borders, to achieve heart health for everyone, everywhere.
